# Supplementary material for: A Meta-Analysis of the Reliability of Four Field-Based Trunk Extension Endurance Tests
Source: Int J Environ Res Public Health. 2020 Apr 29;17(9):3088. doi: 10.3390/ijerph17093088 (PMC7246735; doi:10.3390/ijerph17093088)
Supplement: Supplementary file 1 [file ijerph-17-03088-s001.pdf]

Table S1. The Preferred Reporting Items for Systematic Reviews and Meta-Analyses (PRISMA) checklist.

Section/Topic	#	Checklist Item	Reported on Page #
**TITLE**			
Title	1	Identify the report as a systematic review, meta-analysis, or both.	1
**ABSTRACT**			
Structured summary	2	Provide a structured summary including, as applicable: background; objectives; data sources; study eligibility criteria, participants, and interventions; study appraisal and synthesis methods; results; limitations; conclusions and implications of key findings; systematic review registration number.	1
**INTRODUCTION**			
Rationale	3	Describe the rationale for the review in the context of what is already known.	2–3
Objectives	4	Provide an explicit statement of questions being addressed with reference to participants, interventions, comparisons, outcomes, and study design (PICOS).	3
**METHODS**			
Protocol and registration	5	Indicate if a review protocol exists, if and where it can be accessed (e.g., web address), and, if available, provide registration information including registration number.	3 (PROSPERO ID: CRD42019123179)
Eligibility criteria	6	Specify study characteristics (e.g., PICOS, length of follow-up) and report characteristics (e.g., years considered, language, publication status) used as criteria for eligibility, giving rationale.	3
Information sources	7	Describe all information sources (e.g., databases with dates of coverage, contact with study authors to identify additional studies) in the search and date last searched.	3
Search	8	Present full electronic search strategy for at least one database, including any limits used, such that it could be repeated.	Table S2
Study selection	9	State the process for selecting studies (i.e., screening; eligibility; included in systematic review; and, if applicable, included in the meta-analysis).	3
Data collection process	10	Describe method of data extraction from reports (e.g., piloted forms, independently, in duplicate) and any processes for obtaining and confirming data from investigators.	4 and Box S1
Data items	11	List and define all variables for which data were sought (e.g., PICOS, funding sources) and any assumptions and simplifications made.	4-5

Section/Topic	#	Checklist Item	Reported on Page #
Risk of bias in individual studies	12	Describe methods used for assessing risk of bias of individual studies (including specification of whether this was done at the study or outcome level), and how this information is to be used in any data synthesis.	5 and Table S3
Summary measures	13	State the principal summary measures (e.g., risk ratio, difference in means).	4–5
Synthesis of results	14	Describe the methods of handling data and combining results of studies, if done, including measures of consistency (e.g., I^2) for each meta-analysis.	6
Risk of bias across studies	15	Specify any assessment of risk of bias that may affect the cumulative evidence (e.g., publication bias, selective reporting within studies).	Not applicable
Additional analyses	16	Describe methods of additional analyses (e.g., sensitivity or subgroup analyses, meta-regression), if done, indicating which were pre-specified.	6
**RESULTS**			
Study selection	17	Give numbers of studies screened, assessed for eligibility, and included in the review, with reasons for exclusions at each stage, ideally with a flow diagram.	6<br>Figure 1
Study characteristics	18	For each study, present characteristics for which data were extracted (e.g., study size, PICOS, follow-up period) and provide the citations.	7 and Table S4
Risk of bias within studies	19	Present data on risk of bias of each study and, if available, any outcome level assessment (see item 12).	6–9, Table 1 and Table S5
Results of individual studies	20	For all outcomes considered (benefits or harms), present, for each study: (a) simple summary data for each intervention group, and (b) effect estimates and confidence intervals, ideally with a forest plot.	10<br>Figure 2
Synthesis of results	21	Present results of each meta-analysis performed, including confidence intervals and measures of consistency.	Table 2
Risk of bias across studies	22	Present results of any assessment of risk of bias across studies (see item 15).	Not applicable
Additional analysis	23	Give results of additional analyses, if performed (e.g., sensitivity or subgroup analyses, meta-regression (see item 16)).	11–14<br>Tables 3, 4, and 5
**DISCUSSION**			

Summary of evidence	24	Summarize the main findings, including the strength of evidence for each main outcome; consider their relevance to key groups (e.g., healthcare providers, users, and policymakers).	15–17
Limitations	25	Discuss limitations at the study and outcome level (e.g., risk of bias), and at the review-level (e.g., incomplete retrieval of identified research, reporting bias).	17
Conclusions	26	Provide a general interpretation of the results in the context of other evidence, as well as implications for future research.	17
**FUNDING**			
Funding	27	Describe sources of funding for the systematic review and other support (e.g., supply of data); role of funders for the systematic review.	18

From: Moher, D.; Liberati, A.; Tetzlaff, J.; Altman, D.G.; The PRISMA Group. Preferred Reporting Items for Systematic Reviews and Meta-Analyses: The PRISMA Statement. *PLoS Med* **2009**, *6*, e1000097, doi:10.1371/journal.pmed1000097.

For more information, visit: **www.prisma-statement.org**.

Table S2. Search strategy (all databases).

	Search Details [1]:
**S1**	"Trunk muscle" OR "core muscle" OR "back muscle" OR "trunk extens*".
S2	"Endurance" OR "strength" OR "performance".
S3	S1 AND S2 AND test.
S4	S3 AND reliability.

[1] Limits: English and Spanish.

Table S4. Individual characteristics of each study selected.

a) Inter-Tester Reliability

Biering-Sorensen Test						
**First Author, Year, and Country**	**Participant Characteristics**	**Test Description**	**Measurement Protocol**	**Reliability Coefficient**	**Conclusion** [a]	**Mean Results**
Arab et al. (2007) [18]	30 asymptomatic subjects	Modification of the original version.	Two testers 15 min apart	$ICC_{2,1}$ = 0.78	Poor	35.5 ± 7 s

Iran	(15 females)<br>Mean age: 40.5 years	From the upper border of iliac crest and arms across the chest.<br>Until exhaustion.<br>Visual position control.				
Larsson et al. (2015) [24]<br>Sweden	37 asymptomatic engineer soldiers (4 females)<br>Mean age: 26 years	Modification of the original version.<br>From the ASIS and hands at the level of the ears.<br>Until exhaustion.<br>Visual position control.	Four testers measured at the same time the same group of participants	$ICC_{2,1} = 0.99$	Very good	100.5 ± 29.2 s
Latimer et al. (1999) [23]<br>Australia	(a) 23 adults with current low back pain (LBP) (13 females)<br>Mean age: 35.9 years<br>(b) 20 adults with previous LBP (9 females)<br>Mean age: 36.6 years<br>(c) 20 asymptomatic adults (10 females)<br>Mean age: 28.8 years	Modification of the original version.<br>From the ASIS and arms across the chest.<br>Until exhaustion.<br>Position control with inclinometer.	Two testers<br>15 min apart	a) $ICC_{1,1} = 0.88$ (0.73–0.95)<br>b) $ICC_{1,1} = 0.77$ (0.52–0.9)<br>c) $ICC_{1,1} = 0.83$ (0.62–0.93)	(a) Acceptable<br>(b) Poor<br>(c) Acceptable	(a) 94.6 ± 33.4 s<br>(b) 107.7 ± 36.4 s<br>(c) 132.6 ± 42.2 s
Palacín-Marín et al. (2013) [66]<br>Spain	15 adults with LBP (9 females)<br>Mean age: 37 years	Modification of the original version.<br>From the upper border of iliac crest and arms across the chest.<br>Until exhaustion.<br>Position control with an inclinometer.	One tester examined in a real-time scenario and other tester examined the video recorded	$ICC_{2,1} = 0.92$ (0.91–0.93)	Very good	62.1 ± 32.6 s
Simmonds et al. (1998) [26]<br>United States	(a) 22 adults with LBP (14 females)<br>Mean age: 42.60 years<br>(b) 48 asymptomatic adults (27 females)<br>Mean age: 35.4 years	Modification of the original version.<br>From the upper border of iliac crest and arms along the body.<br>Until exhaustion.	Two testers measured twice within the test session	(a) $ICC_{1,1} = 0.99$<br>(b) $ICC_{1,1} = 0.99$	(a) Very good<br>(b) Very good	(a) 45.9 ± 43 s<br>(b) 77.8 ± 36.7 s

		Visual position control.				
**Prone Isometric Chest Raise Test**						
Arab et al. (2007)<br>[18]<br>Iran	30 asymptomatic adults<br>(15 females)<br>Mean age: 40.5 years	Modification of the original version.<br>Until exhaustion.<br>Visual position control.	Two testers<br>15 min apart	$ICC_{2,1} = 0.9$	Very good	46 ± 13.5 s
**Prone Double Straight-Leg Raise Test**						
Arab et al. (2007)<br>[18]<br>Iran	30 asymptomatic adults<br>(15 females)<br>Mean age: 40.5 years	Original version.<br>Until exhaustion.<br>Visual position control.	Two testers<br>15 min apart	$ICC_{2,1} = 0.83$	Acceptable	36.5 ± 5.5 s

b) Intra-Tester and Intra-Session Reliability

Biering-Sorensen Test						
First Author, Year, and Country	Participant Characteristics	Test Description	Measurement Protocol	Reliability Coefficient	Conclusion [a]	Mean Results
Arab et al. (2007) [18] Iran	30 asymptomatic adults (15 females) Mean age: 40.5 years	Modification of the original version. From the upper border of iliac crest and arms across the chest. Until exhaustion. Visual position control.	Two measures 15 min apart	$ICC_{3,1}$ tester a = 0.8 $ICC_{3,1}$ tester b = 0.79	Tester a = acceptable Tester b = poor	35.5 ± 7 s
Demoulin et al. (2008) [43] Belgium	(a) 10 male university students Mean age: 22.3 years (b) 10 female university students Mean age: 20.7 years (c) 10 male asymptomatic adults Mean age: 41.1 years (d) 10 female asymptomatic adults Mean age: 42 years (e) 10 male adults with LBP Mean age: 39.2 years (f) 10 female adults with LBP Mean age: 45.9 years	Modification of the original version. From the ASIS and arms across the chest. Until exhaustion. Position control with a stadiometer.	Two measures within the same session	(a) ICC_{SEM} = 0.967 (b) ICC_{SEM} = 0.943 (c) ICC_{SEM} = 0.852 (d) ICC_{SEM} = 0.966 (e) ICC_{SEM} = 0.931 (f) ICC_{SEM} = 0.947	(a) Very good (b) Very good (c) Acceptable (d) Very good (e) Very good (f) Very good	(a) 113 ± 27.9 s (b) 141.8 ± 37.5 s (c) 130.8 ± 36.5 s (d) 122.5 ± 27.3 s (e) 93.2 ± 29 s (f) 114.8 ± 27.3 s
Moffroid et al. (1993) [25] United States	28 female asymptomatic undergraduate students Mean age (no info available)	Modification of the original version. Arms along the body. Until exhaustion. Visual position control.	Two measures 15 min apart	ICC_{NIA} = 0.87	Acceptable	190.2 ± 66.3 s
Simmonds et al. (1998) [26]	(a) 44 adults with LBP (28 females)	Not validated modification. From the upper border of iliac	Two measures within the test	(a) $ICC_{1,1}$ = 0.91 (b) $ICC_{1,1}$ = 0.73	(a) Desirable (b) Large	(a) 38.1 ± 34.9 s (b) 120.1 ± 75.3 s

United States	Mean age: 42.6 years<br>(b) 48 asymptomatic adults (27 females)<br>Mean age: 35.8 years	crest and arms along the body.<br>Until exhaustion.<br>Visual position control.	session			
Souza et al. (2016) [67]<br>Brazil	48 female adults with LBP<br>Mean age: 52 years	Validated modification.<br>From the ASIS and arms across the chest.<br>Until exhaustion.<br>Position control with inclinometer.	Two measures 15 min apart	$ICC_{2,1}$ = 0.87	Acceptable	54 ± 36 s
**Prone Isometric Chest Raise Test**						
Arab et al. (2007) [18]<br>Iran	30 asymptomatic adults (15 females)<br>Mean age: 40.5 years	Validated modification.<br>Until exhaustion.<br>Visual position control.	Two measures 15 min apart	$ICC_{3,1}$ tester a = 0.9<br>$ICC_{3,1}$ tester b = 0.89	Tester a = desirable<br>Tester b = acceptable	46 ± 13.5 s
**Prone Double Straight-Leg Raise Test**						
Arab et al. (2007) [18]<br>Iran	30 asymptomatic adults (15 females)<br>Mean age: 40.5 years	Original.<br>Until exhaustion.<br>Visual position control.	Two measures 15 min apart	$ICC_{3,1}$ tester a = 0.87<br>$ICC_{3,1}$ tester b = 0.85	Tester a = acceptable<br>Tester b = acceptable	36.5 ± 5.5 s

c) Intra-Tester and Inter-Session Reliability

Biering-Sorensen Test						
**First Author, Year, and Country**	**Participants Characteristics**	**Test Description**	**Measurement Protocol**	**Reliability Coefficient**	**Conclusion [a]**	**Mean Results**
Dedering et al. (2000) [53] Sweden	10 asymptomatic adults (8 females) Mean age: 28.5 years	Modification of the original version. Hips flexed 40º and arms across the chest. Until exhaustion. Position control with sensor light.	Three measures 1 week apart	$ICC_{3,1}$ = 0.89	Acceptable	303.3 s
Dedering et al. (2010) [54] Sweden	15 adults with lumbar disc herniation (7 females) Mean age: 46 years	Modification of the original version. Hips flexed 40º and arms across the chest. Until exhaustion. Position control with sensor light.	Three measures 1 week apart	$ICC_{3,1}$ = 0.85	Acceptable	206 s
Demoulin et al. (2008) [43] Belgium	a) 10 male university students Mean age: 22.3 years b) 10 female university students Mean age: 20.7 years	Modification of the original version. From the ASIS and arms across the chest. Until exhaustion. Position control with a stadiometer.	Two measures 2 days apart	(a) ICC_{SEM} = 0.98 (b) ICC_{SEM} = 0.92	(a) Desirable (b) Desirable	a) 113 ± 27.9 s b) 141.8 ± 37.5 s
Demoulin et al. (2016) [56] Belgium	40 university students (20 females) Mean age: 21.1 years	Modification of the original version. Arms across the chest. Until exhaustion. Position control with stadiometer.	Two measures 3 days apart	$ICC_{3,1}$ = 0.87	Acceptable	157.2 ± 34.2 s
Geldhof et al. (2007) [57]	47 elementary schoolchildren	Modification of the original version.	Two measures 1 week apart	$ICC_{3,1}$ = 0.63	Large	157.5 ± 53.75 s

Belgium	Mean age: 10.1 years	From the upper border of the iliac crest and hands at the level of the ears. Until 240 seconds. Visual position control.				
Gruther et al. (2009) [8] Austria	21 adults with LBP Mean age: 43 years	Modification of the original version. From the upper border of the iliac crest and arms across the chest. Until exhaustion. Visual position control.	Two measures 2 weeks apart	$ICC_{3,1}$ = 0.59	Large	85.2 ± 49.3 s
Hannibal III et al. (2006) [27] United States	(a) 40 asymptomatic male adolescents Mean age: 15.1 years (b) 32 asymptomatic female adolescents Mean age: 15.5 years	Modification of the original version. Roman chair and arms across the chest. Until exhaustion. Position control with plumb-line.	Two measures Less than 1 week apart	(a) ICC_{ANOVA} = 0.99 (b) ICC_{ANOVA} = 0.99	(a) Desirable (b) Desirable	(a) 146.9 ± 65.1 s (b) 132.0 ± 46.0 s
Jorgensen et al. (1986) [59] Denmark	10 asymptomatic male adults Mean age: 28 years	Modification of the original version. From the upper border of the iliac crest and arms across the chest. Until exhaustion. Visual position control.	Two measures 2 weeks apart	ICC_{NIA} = 0.89	Acceptable	277 ± 60.6 s
Juan-Recio et al. (2014) [60] Spain	27 asymptomatic male adults Mean age: 23.5 years	Modification of the original version. From the ASIS and arms across the chest. Until exhaustion. Visual position control.	Two measures 2 weeks apart	$ICC_{2,1}$ = 0.84 (0.72 - 0.91)	Acceptable	143.4 ± 42.5 s
Juan-Recio et al. (2018) [10]	27 asymptomatic male adults	Modification of the original version.	Two measures 1 month apart	$ICC_{2,1}$ = 0.78 (0.62 - 0.88)	Very large	136.92 ± 41.84 s

Spain	Mean age: 24.1 years	From the ASIS and arms across the chest. Until exhaustion. Position control with stadiometer.				
Keller et al. (2001) [61] Norway	(a) 31 adults with LBP (24 females) Mean age: 36 years (b) 31 asymptomatic adults (24 females) Mean age: 32 years	Modification of the original version. Roman chair, from pubis and arms across the chest. Until exhaustion. Visual position control.	Two measures 2 weeks apart	(a) $ICC_{1,k}$ = 0.93 (b) $ICC_{1,k}$ = 0.8	(a) Desirable (b) Acceptable	(a) 95.5 s (b) 138 s
Larsson et al. (2015) [24] Sweden	20 male ranger soldiers Mean age: 24 years	Modification of the original version. From the ASIS and hands at the level of the ears. Until exhaustion. Visual position control.	Two measures 1 week apart	$ICC_{3,1}$ = 0.85 (0.66–0.94)	Acceptable	100.5 ± 29.2 s
Lin et al. (2013) [62] Taiwan	10 asymptomatic male adolescents Mean age: 16.8 years	Modification of the original version. From the upper border of the iliac crest and arms across the chest. Until exhaustion. Visual position control.	Two measures No further information available	ICC_{NIA} = 0.75	Very large	93.0 ± 29.8 s
Mannion et al. (1997) [63] United Kingdom	10 asymptomatic adults Mean age: 26.10 years	Modification of the original version. From the upper border of the iliac crest and hands at the level of the ears. Until exhaustion. Visual position control.	Two measures 1 day apart	ICC_{ANOVA} = 0.98	Desirable	192.4 ± 76.7 s
Mayer et al. (1995) [9] United States	12 asymptomatic male adults Mean age: 30.20 years	Modification of the original version. Roman chair, from the	Two measures 2 days apart	ICC_{NIA} = 0.2	Poor	No info available

		pubis and arms across the chest.<br>Until exhaustion.<br>Visual position control.				
Moffroid et al. (1994) [64]<br>United States	(a) 32 adults with LBP (18 females)<br>Mean age: 29.3 years<br>(b) 9 Active adults with LBP (4 females)<br>Mean age: 30.5 years<br>(c) 20 inactive adults with LBP (13 females)<br>Mean age: 28.1 years	Modification of the original version.<br>Arms across the chest.<br>Until exhaustion.<br>Position control with inclinometer.	Two measures<br>1 day apart	(a) ICC_{ANOVA} = 0.82<br>(b) ICC_{ANOVA} = 0.96<br>(c) ICC_{ANOVA} = 0.39	(a) Acceptable<br>(b) Desirable<br>(c) Moderate	a) 48.6 ± 34.01 s<br>b) 69.3 ± 59.5 s<br>c) 40.6 ± 14.9 s
Ozcan Kahraman et al. (2016) [65]<br>Turkey	38 adults with LBP (14 females)<br>Mean age: 35 years	Modification of the original version.<br>Arms across the chest.<br>Until exhaustion.<br>Visual position control.	Two measures<br>2 days apart	$ICC_{2,1}$ = 0.88<br>(0.79–0.93)	Acceptable	32.2 ± 24.6 s
Palacín-Marín et al. (2013) [66]<br>Spain	15 adults with LBP (9 females)<br>Mean age: 37 years	Modification of the original version.<br>From the upper border of iliac crest and arms across the chest.<br>Until exhaustion.<br>Position control with an inclinometer.	The same tester examined the video recording at 1 month after the first assessment	$ICC_{2,1}$ = 0.94<br>(0.93–0.95)	Desirable	62.1 ± 32.6 s
Simmonds et al. (1998) [26]<br>United States	(a) 44 adults with LBP (28 females)<br>Mean age: 42.60 years<br>(b) 48 asymptomatic adults (27 females)<br>Mean age: 35.4 years	Modification of the original version.<br>From the upper border of iliac crest and arms along the body.<br>Until exhaustion.<br>Visual position control.	Two measures<br>2 weeks apart	(a) $ICC_{1,1}$ = 0.88<br>(b) $ICC_{1,1}$ = 0.68	(a) Acceptable<br>(b) Very large	a) 38.1 ± 34.9 s<br>b) 120.1 ± 75.3 s
Teyhen et al. (2011)	64 asymptomatic adults	Modification of the original	Two measures	$ICC_{2,1}$ = 0.79	Very large	108.6 ± 39.6 s

[68]<br>United States	(11 females)<br>Mean age: 25.2 years	version.<br>From the ASIS and arms across the chest.<br>Until 240 seconds.<br>Position control with inclinometer.	2 days apart	(0.67–0.87)		
Waldhelm and Li (2012) [69]<br>United States	15 asymptomatic male undergraduate students<br>Mean age: 21.20 years	Modification of the original version.<br>Arms across the chest.<br>Until exhaustion.<br>Visual position control.	Two measures<br>1 week apart	$ICC_{2,1}$ = 0.79<br>(0.38–0.93)	Very large	83.3 ± 29.4 s
**Dynamic Extensor Endurance Test**						
Hannibal III et al., (2006) [27]<br>United States	(a) 40 asymptomatic male adolescents<br>Mean age: 15.1 years<br>(b) 32 asymptomatic female adolescents<br>Mean age: 15.5 years	(1) Modification of the original version.<br>Roman chair, ROM of 90º and arms across the chest.<br>Until exhaustion, 20 rep/min.<br>Position control with plumb-line.<br>(2) Modification of the original version.<br>ROM of 90º and arms across the chest.<br>Until exhaustion, 20 rep/min.<br>Visual position control.	Two measures<br>Less than 1 week apart	(1a) ICC_{ANOVA} = 0.99<br>(1b) ICC_{ANOVA} = 0.99<br>(2a) ICC_{ANOVA} = 0.99<br>(2b) ICC_{ANOVA} = 0.99	(1a) Desirable<br>(1b) Desirable<br>(2a) Desirable<br>(2b) Desirable	(1a) 57.1 ± 34.3 reps<br>(1b) 50.6 ± 22.6 reps<br>(2a) 53.4 ± 29.9 reps<br>(2b) 44.0 ± 19.8 reps
Lin et al. (2013) [62]<br>Taiwan	10 asymptomatic male adolescents<br>Mean age: 16.8 years	Modification of the original version.<br>ROM of 30º and arms across the chest.<br>Until exhaustion.<br>Visual position control.	Two measures<br>1 week apart	ICC_{NIA} = 0.92	Desirable	27.0 ± 10.3 reps
**Prone Isometric Chest Raise Test**						

del Pozo-Cruz et al. (2014) [55] Spain	(a) 12 male adults with LBP Mean age: 45.8 years (b) 19 female adults with LBP Mean age: 46 years	Modification of the original version. Until exhaustion. Visual position control.	Two measures 1 week apart	(a) ICC_{NIA} = 0.97 (b) ICC_{NIA} = 0.96	(a) Desirable (b) Desirable	(a) 82.4 ± 25.2 s (b) 84.3 ± 31.1 s
Ito et al. (1996) [11] Japan	(a) 90 asymptomatic adults (53 females) Mean age: 45.5 years (b) 100 adults with LBP (60 females) Mean age: 45.4 years	Original version. Until 300 seconds. Visual position control.	Two measures 3 days apart	(a) ICC_{NIA} = 0.97 (b) ICC_{NIA} = 0.93	(a) Desirable (b) Desirable	(a) 160.3 ± 70.5 s (b) 76.3 ± 53.9 s
Javadian et al. (2012) [58] Iran	15 adults with LBP Range age 18 - 45 years	Original version. Until 300 seconds. Visual position control.	Two measures 2 days apart	ICC_{NIA} = 0.85	Acceptable	22.5 ± 4.5 s

Biering-Sorensen test (Biering-Sorensen, 1984) is evaluated by measuring how many seconds the participant was able to keep the unsupported upper part of the body (from the upper border of the iliac crest) horizontal while placed prone with the buttocks and legs fixed to the table bench by three wide canvas straps, and the arms across the chest. The test is continued until the participant could no longer control his/her posture for a maximum of 240 s. Prone isometric chest raise test (Ito et al., 1996) is assessed by measuring how many seconds the participant was able to keep the sternum off the floor while placed lying in a prone position, with the arms along the body. A small pillow is placed under the iliac crest to decrease the lumbar lordosis. The subject is asked to maintain the positions for as long as possible, not exceeding a 5 min time limit. Prone double straight-leg raise test (McIntosh, Wilson, Affleck, and Hall, 1998) is evaluated with the participant in a prone position with hips extended, the hands underneath the forehead, and the arms perpendicular to the body. The participant is instructed to raise both legs until knee clearance is achieved. The test is continued until the participant could no longer able to maintain knee clearance. Dynamic extensor endurance test (Luoto, Heliövaara, Hurri, and Alaranta, 1995) is performed with the subject in a prone position with the unsupported upper part of the body (from the upper border of the iliac crest). The arms are positioned along the body and the buttocks and legs are fixed by three straps. With the spine kept straight, the subject is instructed to extend the trunk to neutral and then to lower the upper body 45 degrees. A repeated beat guided the subject to maintain a cadence of 25 repetitions per minute until exhaustion. The number of repetitions accomplished by the subject is counted. [a] ICC values were interpreted according to the following criteria: <0.1, trivial; 0.1–0.29, small; 0.3–0.49, moderate; 0.5–0.69, large; 0.7–0.89, very large; 0.9–1, nearly perfect. (a–f) indicate different cohorts in the same study, ASIS: anterior superior iliac spine, ROM: range of motion, $ICC_{2,1}$: intraclass correlation coefficient based on two-way random effects model, $ICC_{1,1}$: intraclass correlation coefficient based on one-way random effects model, $ICC_{1,k}$: intraclass correlation coefficient based on one-way random effects model, $ICC_{3,1}$: intraclass correlation coefficient based on two-way mixed effects model, ICC_{NIA}: no information available about intraclass correlation coefficient calculation, ICC_{ANOVA}: intraclass correlation coefficient calculated from results obtained from analysis of variance (ANOVA), ICC_{SEM}: intraclass correlation coefficient calculated from standard error of measurement scores and using standardized equations (see method section for more information),s: seconds, reps: repetitions.

Box S1. Coding protocol.

General Study Descriptors
▪ Authors.
▪ Publication year.
▪ Language.
▪ Tester background (sports sciences, physical therapy, or medicine).
▪ Conflict of interest (yes or no).
▪ Continent (Europe, Africa, Asia, America, Oceania, Antarctica).
▪ Study objective (psychometric or not psychometric).
▪ Reliability analysis done with the same sample (yes or no).
**Description of the Study Population**
▪ Initial sample size.
▪ Final sample size.
▪ Sample type (children and adolescents or adults).
▪ Age and standard deviation.
▪ Gender distribution (% female).
▪ Gender (males, females, or males and females).
▪ Target population (asymptomatic or clinical).
▪ Physical activity level (sedentary, recreationally active, or athlete).
▪ Percentage attrition.
**Description of the Field-Based Tests**
▪ Test (Biering-Sorensen test, prone isometric chest raise test, prone double straight-leg raise test, or dynamic extensor endurance test).
▪ Test version (original or modified).
▪ Validated modification (yes or no).
▪ Tool (test bench or roman chair).
▪ Hand position (crossed on the chest, along the body, at the level of the ears).
▪ Part of the body on the edge (upper border of the iliac crest, anterior superior iliac spine (ASIS), not reported).
▪ Test duration (exhaustion or stopped in a certain time).
▪ Cadence (if it is applicable).
▪ Position control systems (visually, inclinometer, stadiometer, light sensor, plumb-line).
▪ Test score from the total sample (mean and SD).
▪ Test score from the reliability sample (mean and SD).
**Type of Reliability Analysis**
▪ Reliability (inter-tester or intra-tester (intra-session or internal consistency or inter-session or stability)).
▪ Intraclass correlation coefficient.
**Characteristics of the Study Design**
▪ Familiarization session (yes or no).
▪ Number of measurements.
▪ Number of testers.
▪ Time interval between measurements.
▪ Test conditions (type of administration, environment, instructions, etc. (yes or no)).

Table S3. Description of the consensus-based standards for the selection of health measurement instruments (COSMIN) methodology.

Steps	Description

**1. COSMIN risk of bias check-list**	The checklist contains standards referring to design requirements and preferred statistical methods of studies on measurement properties. For each measurement property, a COSMIN box was developed containing all standards needed to assess the quality of a study on that specific measurement property. Each standard of the box is rated as "very good", "adequate", "doubtful", or "inadequate" quality. The overall rating of the quality of each study is determined by taking the lowest rating of any standard in the box.
**2. Criteria for good measurement properties**	The results of each study on a measurement property should be rated against the criteria for good measurement properties. Each result is rated as either sufficient (+ = ICC ≥ 0.70), insufficient (− = ICC < 0.70), or indeterminate (? = ICC not reported).
**3. Summarize the evidence and grade the quality of the evidence**	The results from different studies on one measurement property can be quantitatively pooled in a meta-analysis or qualitatively summarized. After pooling or summarizing all evidence per measurement property, and rating the pooled or summarized results against to the criteria for good measurement properties, the quality of the evidence is graded (high, moderate, low, very low evidence) on the basis of the Grading of Recommendations Assessment, Development and Evaluation (GRADE) approach.<br>The GRADE approach uses five factors to determine the quality of the evidence: risk of bias (i.e., the methodological quality of the studies), inconsistency (i.e., unexplained inconsistency of results across studies), indirectness (i.e., evidence from different populations, interventions, or outcomes than the ones of interest), imprecision (i.e., total sample size of the available studies), and publication bias (i.e., negative results are less often published). The fifth factor, publication bias, is difficult to assess in studies on measurement properties, because of a lack of registries for this type of studies. Therefore, we do not take this factor into account in this meta-analysis [40].

Table S5. COSMIN risk of bias checklist (Box 6 reliability) and criteria for good measurement properties.

d) Inter-Tester Reliability

**Biering-Sorensen Test**							
**Study**	**Standards for Assessing the Risk of Bias**					**Final Score**	**Good Measurement Properties**
	**1**	**2**	**3**	**4**	**8**		
Arab et al., 2007 [18]	VG	D	A	VG	VG	D	+
Larsson et al., 2015 [24]	VG	VG	VG	VG	VG	VG	+
Latimer et al., 1999 [23]	A	D	A	VG	D	D	+
Palacín-Marín et al., 2013 [66]	VG	VG	VG	VG	VG	VG	+
Simmonds et al., 1998 [26]	A	D	A	VG	VG	D	+
**Prone Isometric Chest Raise Test**							

Arab et al., 2007 [18]	VG	D	A	VG	VG	D	+
**Prone Double Straight-Leg Raise Test**							
Arab et al., 2007 [18]	VG	D	A	VG	VG	D	+

e) Intra-Tester Reliability and Intra-Session Reliability

**Biering-Sorensen Test**							
**Study**	**Standards for Assessing the Risk of Bias**					**Final Score**	**Good Measurement Properties**
	**1**	**2**	**3**	**4**	**8**		
Arab et al., 2007 [18]	VG	D	A	VG	VG	D	+
Demoulin et al., 2008 [43]	A	D	A	VG	D	D	+
Moffroid et al., 1993 [25]	A	D	A	A	D	D	+
Simmonds et al., 1998 [26]	A	D	A	VG	VG	D	+
Souza et al., 2016 [67]	A	D	A	A	D	D	+
**Prone Isometric Chest Raise Test**							
Arab et al., 2007 [18]	VG	D	A	VG	VG	D	+
**Prone Double Straight-Leg Raise Test**							
Arab et al., 2007 [18]	VG	D	A	VG	VG	D	+

f) Intra-Tester Reliability and Inter-Session Reliability

**Biering-Sorensen Test**							
**Study**	**Standards for Assessing the Risk of Bias**					**Final Score**	**Good Measurement Properties**
	**1**	**2**	**3**	**4**	**8**		
Dedering et al., 2000 [53]	VG	D	VG	VG	VG	D	+
Dedering et al., 2010 [54]	VG	D	VG	VG	VG	D	+
Demoulin et al., 2008 [43]	A	D	A	VG	D	D	+
Demoulin et al., 2016 [56]	VG	D	VG	VG	VG	D	+
Geldhof et al., 2007 [57]	VG	D	VG	VG	VG	D	−
Gruther et al., 2009 [8]	VG	VG	VG	VG	VG	VG	−
Hannibal III et al., 2006 [27]	A	D	A	VG	D	D	+
Jorgensen & Nicolaisen 1986 [59]	VG	VG	VG	VG	VG	VG	+
Juan-Recio et al., 2014 [60]	VG	D	VG	VG	VG	D	+
Juan-Recio et al., 2018 [10]	VG	VG	VG	VG	VG	VG	+
Keller et al., 2001 [61]	VG	VG	VG	VG	VG	VG	+
Larsson et al., 2015 [24]	VG	D	VG	VG	VG	D	+
Lin et al., 2013 [62]	A	D	A	A	I	I	+
Mannion et al., 1997 [63]	A	D	A	VG	I	I	+
Mayer et al., 1995 [9]	A	D	A	A	D	D	−
Moffroid et al., 1994 [64]	VG	D	VG	VG	D	D	−
Ozkan Kahraman et al., 2016 [65]	VG	D	VG	VG	D	D	+
Palacín-Marín et al., 2013 [66]	VG	VG	VG	VG	VG	VG	+
Simmonds et al., 1998 [26]	A	VG	A	VG	VG	A	+
Teyhen et al., 2011 [68]	A	D	A	VG	D	D	+
Waldhelm & Li 2012 [69]	A	D	A	VG	D	D	+
**Dynamic Extensor Endurance Test**							

Hannibal III et al., 2006 [27]	A	D	A	VG	D	D	+
Lin et al., 2013 [62]	A	D	A	A	I	I	+
**Prone Isometric Chest Raise Test**							
del Pozo-Cruz et al., 2014 [55]	A	D	A	D	D	D	+
Ito et al., 1996 [11]	A	D	A	A	D	D	+
Javadian et al., 2012 [58]	A	D	A	A	D	D	+

Standards for assessing the risk of bias:

1. Patients were stable in the interim period on the construct to be measured (very good (VG): evidence provided that patients were stable; adequate (A): assumable that patients were stable; doubtful (D): unclear if patients were stable; inadequate (I): patients were not stable).
2. The time interval was appropriate (VG: time interval appropriate; D: doubtful whether time interval was appropriate or time interval was not stated; I: time interval not appropriate).
3. The test conditions were similar for the measurements (e.g., type of administration, environment, instructions) (VG: test conditions were similar (evidence provided); A: assumable that test conditions were similar; D: unclear if test conditions were similar; I: test conditions were not similar).
4. For continuous scores: an intraclass correlation coefficient (ICC) was calculated (VG: ICC calculated and model or formula of the ICC is described; A: ICC calculated but model or formula of the ICC not described or not optimal. Pearson or Spearman correlation coefficient calculated with evidence provided that no systematic change occurred; D: Pearson or Spearman correlation coefficient calculated without evidence provided that no systematic change occurred or with evidence that systematic change occurred; I: no ICC or Pearson or Spearman correlations calculated).
8. There were any other important flaws in the design or statistical methods of the study (VG: no other important methodological flaws; D: other minor methodological flaws; I: other important methodological flaws).

Criteria for good measurement properties (reliability):

(+) ICC ≥ 0.70

(-) ICC < 0.70

(?) ICC not reported
